# The Signs, Disorders, and Management of Pregnancy; the Treatment to Be Adopted during and after Confinement; and the Management and Disorders of Children. Written Expressly for the Use of Females

**Published:** 1834-07-01

**Authors:** 


*The Signs, Disorders, and Management of Pregnancy; the Treatment to be adopted during and after Confinement; and the Management and Disorders of Children. Written expressly for the Use of Females.* By Douglas Fox, M.R.C.S.—Derby and London, 1834. 8vo. pp. 207.

No medical practitioner can require to be informed how useless, how injurious, are books like the one now before us; yet they find readers, as the fox finds geese. All popular treatises on medicine, and, we regret to add, too many of the vade-mecums and abstracts written for beginners, fall into the same error: they suppose it to be easy to distinguish diseases, and difficult to prescribe for them; the reverse being the truth. Medicine chests, clyster pipes, and medical books, should be eschewed by all laymen who wish to live long and cheerfully; and, if the goodman of the house finds his wife reading a treatise on pregnancy, even though "written expressly for the use of females," we should recommend him to burn it without a moment's hesitation.

